# A Lucknolide Derivative Induces Mitochondrial ROS-Mediated G2/M Arrest and Apoptotic Cell Death in B16F10 Mouse Melanoma Cells

**DOI:** 10.3390/md22120533

**Published:** 2024-11-28

**Authors:** Jae Hyeop Lee, Byeoung-Kyu Choi, Minsoo Kim, Hee Jae Shin, Sun Joo Park

**Affiliations:** 1 BB21 Plus Program, Department of Chemistry, Pukyong National University, Busan 48513, Republic of Korea; woguq2410@gmail.com; 2 Department of Bio-Convergence Engineering, Dongyang Mirae University, Seoul 08221, Republic of Korea; choibk4404@dongyang.ac.kr; 3 Laboratory of Integrative Molecular Medicine, Graduate School of Medicine, Kyoto University, Yoshida-Konoe-cho, Sakyo-ku, Kyoto-shi 606-8501, Japan; kim.minsoo.7r@kyoto-u.ac.jp; 4 Marine Natural Products Laboratory, Korea Institute of Ocean Science and Technology, 385 Haeyang-ro, Yeongdo-gu, Busan 49111, Republic of Korea

**Keywords:** lucknolide derivative, marine Streptomyces, melanoma, mitochondrial ROS, apoptosis, anti-tumor

## Abstract

Melanoma is an aggressive skin cancer with a high risk of cancer-related deaths, and inducing apoptosis in melanoma cells is a promising therapeutic strategy. This study investigates the anti-tumor potential of a novel lucknolide derivative LA-UC as a therapeutic candidate for melanoma. Lucknolide A (LA), a tricyclic ketal-lactone metabolite isolated from marine-derived *Streptomyces* sp., was chemically modified by introducing a 10-undecenoyl group to synthesize LA-UC. LA-UC preferentially inhibited the proliferation of melanoma cells, including B16F10, while exerting minimal effects on normal melanocytes or other tumor cell types, indicating the selective action of LA-UC against melanoma cells. LA-UC decreased G2/M checkpoint proteins, including cyclin B1 and Cdc2, while activating caspase-3 and caspase-9, resulting in G2/M cell cycle arrest and inducing apoptotic cell death in B16F10 cells. The addition of a pan-caspase inhibitor confirmed the caspase-dependent mechanism of LA-UC-induced cell death. Additionally, LA-UC elevated mitochondrial ROS levels, leading to mitochondrial membrane disruption, upregulation of pro-apoptotic proteins, and DNA damage in melanoma cells. The ROS scavenger N-acetylcysteine reduced LA-UC-induced mitochondrial ROS accumulation, mitochondrial membrane disruption, DNA damage, and apoptosis. Collectively, these findings suggest that LA-UC induces G2/M cell cycle arrest and caspase-dependent apoptosis in B16F10 cells through excessive mitochondrial ROS generation, membrane impairment, and DNA damage, highlighting its potential as a promising therapeutic candidate for melanoma treatment.

## 1. Introduction

Melanoma is a malignant skin cancer originating from melanocytes. It accounts for approximately 80% of all skin tumor-related deaths and has a poor prognosis owing to its low sensitivity and resistance to conventional chemotherapy [1]. Therefore, strategies to overcome low drug sensitivity and resistance are promising approaches for melanoma treatment [2,3].

Inducing apoptosis in melanoma cells has emerged as a viable therapeutic strategy because it can directly eliminate tumor cells with minimal impact on normal tissues [4,5]. Given melanoma’s aggressive growth and resistance to treatment, triggering apoptosis offers a targeted approach to inhibiting tumor progression [6]. Melanoma cell apoptosis is mainly induced by the intrinsic mitochondrial pathway [7]. The mitochondrial pathway is primarily regulated by mitochondrial function and is initiated by internal stimuli such as DNA damage, oxidative stress, or oncogene activation. Mitochondrial dysfunction, particularly via the accumulation of mitochondrial reactive oxygen species (ROS), plays a key role in activating apoptosis in melanoma [8,9]. Compounds that increase mitochondrial ROS production induce apoptosis by promoting mitochondrial membrane depolarization, cytochrome c release, and subsequent caspase activation [10,11,12]. Therefore, therapeutic agents that enhance mitochondrial ROS generation and exploit mitochondrial dysfunction have a significant potential for melanoma treatment.

Marine ecosystems rich in diverse and unique microorganisms are valuable sources of novel bioactive compounds with therapeutic potential [13]. Marine-derived compounds often possess distinct chemical structures and mechanisms of action that differ from terrestrial sources [14]. Recent studies have identified marine microorganisms as producers of potent anti-tumor agents capable of modulating key cellular processes such as ROS generation and apoptosis [15]. However, most of these compounds have been less successful in drug development owing to their toxicity and unclear mechanisms of action [16,17,18].

In this study, we report the isolation of lucknolide A (LA) from a marine-derived *Streptomyces* sp. and the semi-synthesis of a novel lucknolide derivative (LA-UC). LA was previously isolated from a terrestrial *Streptomyces* species [19]; however, neither its structural derivatives nor its biological activities have been reported to date. This may be due to LA being a distinctive tricyclic ketal-lactone metabolite. The fused 5/5/6-tricyclic system of LA, with all-cis-ring junction stereochemistry, represents a highly strained structure rarely found in nature. The development of synthetic methods targeting such compounds is particularly challenging [20,21,22]. Therefore, our study serves as the first significant report on the isolation of a highly strained tricycle ketal-lactone compound from a marine organism and its novel biological activity. Our study aimed to investigate the anti-tumor effects of LA and a newly synthesized LA-UC, to elucidate their molecular mechanisms of action, and evaluate their potential as a therapeutic candidate for melanoma.

## 2. Results

### 2.1. Isolation and Structural Elucidation of Lucknolide A (LA)

**Figure 1:**
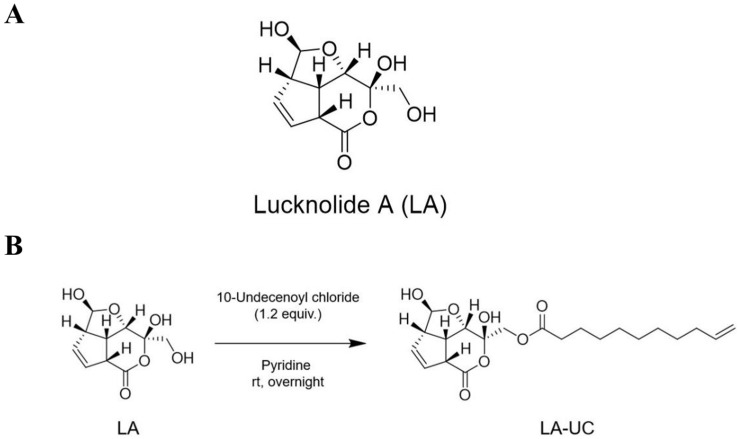
Structures of lucknolide A (LA) and lucknolide derivative (LA-UC), and its synthesis scheme. (**A**) The structure of LA, a natural product isolated from the marine-derived *Streptomyces* sp. strain 151KO-065. (**B**) The structure and its synthesis scheme of LA-UC, a semi-synthetic derivative of LA, synthesized by introducing a 10-undecenoyl group through semi-synthesis.

The producing strain 151KO-065 was isolated from a mangrove sample collected offshore of Kosrae Island and identified as *Streptomyces* sp. by 16S rRNA gene sequence analysis. Large-scale fermentation was conducted to isolate the active components. The culture broth was subjected to solvent extraction and subsequent fractionation using flash column chromatography. After analyzing the NMR spectra of the fractions, a fraction showing unusual peaks in the ^1^H NMR spectrum was selected for further purification using a reversed-phase HPLC. As a result, lucknolide A (LA) was successfully purified (Figure 1A).

LA was obtained as a white powder and gave an [M − H]^−^ ion peak at *m/z* 227.17 (calcd 227.063) in the HRESIMS (Figure S1), consistent with a molecular formula of C_10_H_12_O_6_. The ^1^H and ^13^C NMR data (Figure S2) and HSQC spectra (Figure S3) of LA indicated the presence of one oxygenated methylene (δ_H_ 3.53), five methines (δ_H_ 3.21, 3.23, 3.57, 4.38, 4.96), two olefins (δ_H_ 5.77, 5.89), and two quaternary carbons (δ_C_ 102.5, 171.0). Comprehensive analysis of the ^1^H-^1^H COSY (Figure S4) and HMBC spectra (Figure S5), in conjunction with a detailed literature comparison, allowed us to conclusively identify the isolated compound as LA (Figure 1A). In this study, we aimed to investigate the anti-tumor activity of LA and further improve its potential through structural modification.

### 2.2. Structural Modification of LA: Synthesis of LA-UC

We synthesized an LA derivative containing a 10-undecenoyl group through semi-synthesis because 10-undecenoic acid is a naturally occurring unsaturated fatty acid with a terminal double bond and is often used to enhance a compound’s lipophilicity and improve its cellular membrane permeability, which can potentially increase its biological activity [23,24,25].

LA was reacted with pyridine and 10-undecenoyl chloride at room temperature overnight. After completion of the reaction, the product was purified using open column chromatography. The structure of the purified product was confirmed by analysis of MS and NMR data (Figures S6 and S7). The spectroscopic data verified the successful introduction of the 10-undecenoyl group at the primary alcohol position of LA, leading to the formation of a new lucknolide derivative, LA-UC (Figure 1B).

### 2.3. LA-UC Preferentially Decreases Cell Proliferation of Melanoma Cells

**Figure 2:**
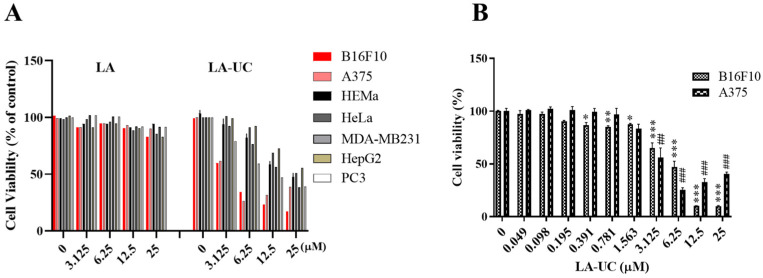
LA-UC suppresses the proliferation of B16F10 mouse melanoma cells. (**A**) Several tumor cell lines, including B16F10, A375, Hela, HepG2, MDA-MB231, PC3, and HEMa human primary melanocytes were treated with LA or LA-UC for 24 h, and the cell viability was assessed by MTT assay. (**B**) B16F10 and A375 cells were treated with various concentrations of LA-UC for 24 h. Results are presented as mean ± standard error of the mean (SEM) of triplicate independent experiments. * *p* < 0.05, **, ^##^ *p* < 0.01, and ***, ^###^ *p* < 0.001, compared to vehicle DMSO control in B16F10 or A375 cells.

To examine the anti-tumor effects of LA and LA-UC, we treated several tumor cell lines, including B16F10, A375, HeLa, HepG2, MDA-MB231, and PC3, with LA or LA-UC for 24 h, and cell viability was measured using the 3-(4,5-dimethylthiazol-2-yl)-2,5-diphenyltetrazolium bromide (MTT) assay. Despite its unique structure, LA did not exhibit anti-tumor activity (Figure 2A). However, the introduction of the 10-undecenoyl group into LA induced weak anti-tumor activity. Notably, we found that LA-UC preferentially suppressed the growth of melanoma cells, B16F10 and A375, compared to other tumor cell types. To further investigate the effect of LA-UC on melanoma cell proliferation, B16F10 and A375 cells were treated with various concentrations (0–25 μM) of LA-UC for 24 h. The IC_50_ value of LA-UC was determined to be approximately 5.3 μM in both melanoma cells (Figure 2B). Moreover, LA-UC exhibited minimal cytotoxicity toward HEMa human normal melanocytes. The IC_50_ concentrations effective against melanoma cells had little effect on HEMa, indicating that LA-UC preferentially targets melanoma cells (Figure 2A).

### 2.4. LA-UC Induces G2/M Phase Arrest and Caspase-Dependent Apoptosis in B16F10 Cells

**Figure 3:**
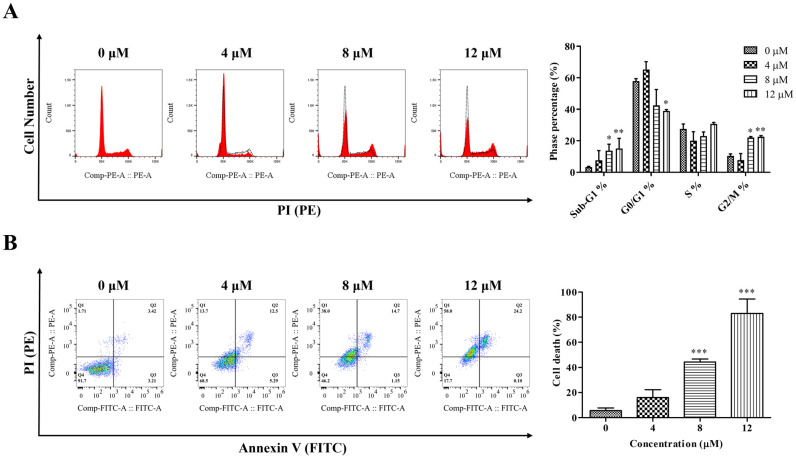
LA-UC induces G2/M arrest and apoptosis in B16F10 mouse melanoma cells. (**A**) Representative cell cycle analysis images showing LA-UC-induced G2/M phase arrest. B16F10 cells were treated with indicated concentrations of LA-UC for 24 h, fixed with ethanol, stained with propidium iodide (PI), and then analyzed using flow cytometry. The black dot line is vehicle DMSO control, and the red line (filled) is LA-UC-treated cells (**B**) Representative fluorescence-activated cell sorting (FACS) images showing LA-UC-induced apoptosis. Cell death was calculated as Annexin V and/or PI-positive cells. Results were quantified and are presented as mean ± standard error of the mean (SEM) of triplicate independent experiments. * *p* < 0.05, ** *p* < 0.01, and *** *p* < 0.001, compared to the control.

**Figure 4:**
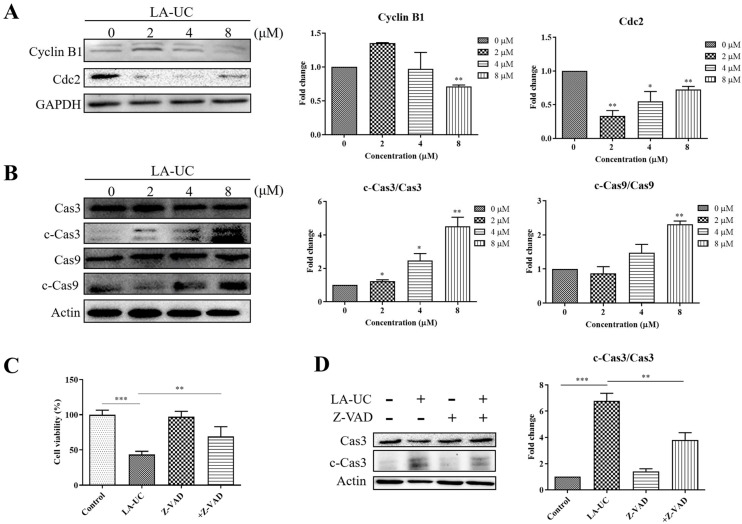
Representative western blot images indicating LA-UC-induced G2/M phase arrest (**A**) and caspase-dependent apoptosis (**B**,**C**). B16F10 cells were treated with indicated concentrations of LA-UC for 24 h. Cyclin B1, Cdc2, caspase-3 and 9, cleaved caspase-3 and 9, GAPDH, and actin expressions were analyzed via immunoblotting. Protein levels were normalized to GAPDH or actin, and the fold change was calculated by dividing the normalized expression levels of each sample by the control value. (**C**,**D**) Cells were treated with Z-VAD-FMK (20 μM) for 12 h and then additionally treated with 8 μM LA-UC for 24 h. (**C**) MTT assay was performed to determine cell viability, and activation of caspase-3 was detected via Western blot analysis (**D**). Results were quantified using Image J and are presented as mean ± standard error of the mean (SEM) of triplicate independent experiments. * *p* < 0.05, ** *p* < 0.01, and *** *p* < 0.001, compared to vehicle control. C-Cas3: cleaved caspase-3; C-Cas9: cleaved caspase-9.

To analyze the mechanisms underlying LA-UC-induced cell growth inhibition in B16F10 cells, we treated the cells with varying concentrations of LA-UC for 24 h, stained with propidium iodide (PI), and analyzed cell cycle progression using flow cytometry. LA-UC arrested cell cycle progression in the G2/M phase in a concentration-dependent manner (Figure 3A). Compared with vehicle-treated control cells, LA-UC treatment increased the number of sub-G1 dead cells and G2/M phase cells and decreased the number of G0/G1 phase cells in a concentration-dependent manner. These results suggest that LA-UC decreased cell cycle progression through cell cycle arrest in the G2/M phase. Moreover, double staining with annexin V and PI showed that LA-UC treatment increased both annexin V-positive and PI-positive cells in a dose-dependent manner (Figure 3B), indicating that LA-UC induces apoptotic cell death. The effect of LA-UC on G2/M phase arrest and apoptosis was further determined by analyzing the expression levels of key proteins responsible for G2/M phase transition and apoptotic signaling. Figure 4A,B shows that LA-UC treatment decreased the expression of cyclin B1 and cell division cycle 2 (Cdc2), which are essential for the transition from G2 to M phase, whereas it activated caspase-3 and caspase-9, key proteins in the caspase-dependent apoptosis pathway. Increased levels of cleaved caspase-3 and caspase-9 were observed in the cells treated with LA-UC. These results suggest that LA-UC-induced cell death was dependent on caspase-dependent apoptosis. Moreover, we treated the cells with LA-UC in the presence or absence of the pan-caspase inhibitor Z-Val-Ala-Asp (OMe)-fluoromethylketone (Z-VAD-FMK [Z-VAD]). Z-VAD treatment reduced LA-UC-induced cell death (Figure 4C) and caspase-3 activation (Figure 4D), indicating that LA-UC induces caspase-mediated apoptosis, which decreases the growth of B16F10 cells.

### 2.5. LA-UC Causes Excessive Mitochondrial ROS Production, Loss of Mitochondrial Membrane Potential, and DNA Damage in B16F10 Cells

**Figure 5:**
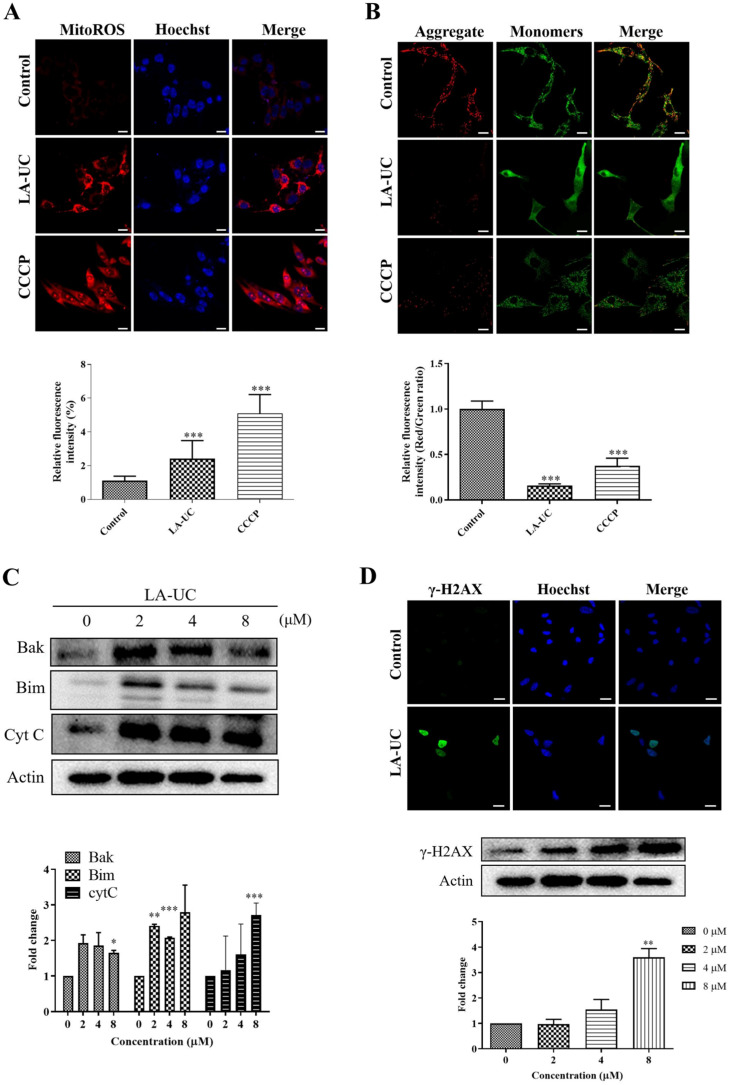
LA-UC induces an increase in mitochondrial ROS, loss of mitochondrial membrane potential, and DNA damage. (**A**) B16F10 cells were treated with 8 μM LA-UC or 15 μM CCCP, a mitochondrial uncoupler, for 12 h. The level of mitochondrial ROS was measured with MitoSOX^TM^ staining, and DNA with Hoechst 33342 staining. Scale bar, 20 μm. (**B**) Levels of mitochondrial membrane potential were measured via JC-1 staining. Representative images are shown. The intensity ratio of the green-to-red fluorescence was determined. Data are presented as the mean ± standard error of the mean (SEM). (**C**) Cells were treated the same as in (**A**,**B**), and expression levels of Bak, Bim, and cytochrome C were detected. (**D**) γ-H2AX protein was visualized via immunofluorescence using its primary antibodies to detect DNA damage and also determined by western blotting. Scale bar, 20 μm. Results were quantified and are presented as mean ± SEM of triplicate independent experiments. * *p* < 0.05, ** *p* < 0.01, and *** *p* < 0.001, compared to control. Cyt C: Cytochrome C.

Next, we explored the underlying mechanism by which LA-UC induces G2/M phase cell cycle arrest and apoptosis in B16F10 cells. ROS are signaling mediators of normal cellular function; however, abnormally excessive production and accumulation lead to oxidative imbalance, resulting in DNA damage, cell cycle arrest, and apoptosis [26]. Impairment of the ROS-mediated mitochondrial membrane is a primary apoptotic process [27]. Therefore, we investigated whether LA-UC triggers an increase in mitochondrial ROS and affects the mitochondrial membrane potential in B16F10 cells using MitoSOX^TM^ and JC-1 staining. LA-UC significantly increased the mitochondrial ROS levels and caused mitochondrial membrane potential damage (Figure 5A,B). JC-1, a membrane potential-dependent fluorescent dye, accumulates in healthy mitochondria to form red fluorescent aggregates; however, upon depolarization, these aggregates primarily appear as green fluorescent monomers. In LA-UC-treated cells, JC-1 was present in its monomeric state, indicating impaired mitochondrial membrane potential (Figure 5B). In addition, LA-UC induced the activation of mitochondria-associated pro-apoptotic proteins, Bcl-2 homologous antagonist/killer (Bak), Bcl-2 interacting mediator of cell death (Bim), and cytochrome C (Figure 5C). These results suggest that LA-UC induces cell death through the activation of the intrinsic mitochondrial apoptotic pathway. Immunofluorescence and immunoblot analysis for γ- phosphohistone (H2AX), a DNA damage marker, revealed that the γ-H2A level was increased in cells treated with LA-UC (Figure 5D). These findings indicate that LA-UC induces the excessive production of mitochondrial ROS, impairment of mitochondrial membrane potential, activation of mitochondrial pro-apoptotic proteins, and DNA damage.

### 2.6. Mitochondrial ROS Increase Contributes to LA-UC-Induced Cell Cycle Arrest and Apoptosis in B16F10 Cells

**Figure 6:**
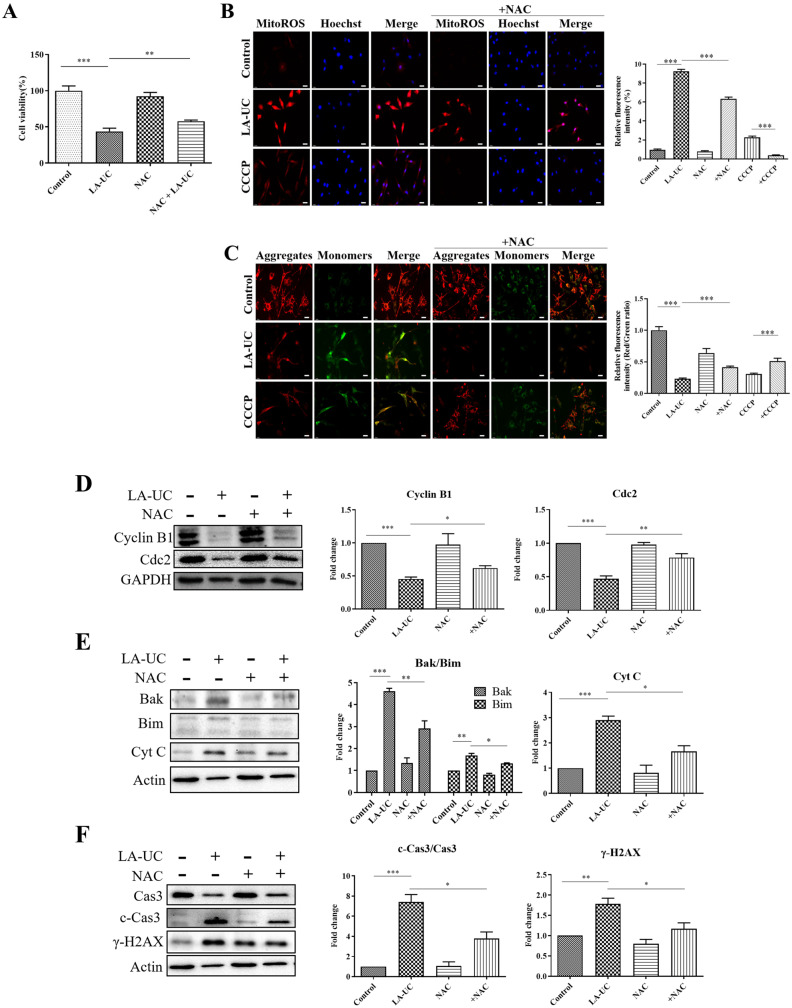
LA-UC-induced excessive generation of mitochondrial ROS is associated with LA-UC-induced cell cycle arrest and apoptosis in B16F10 cells. (**A**–**C**) B16F10 cells were pre-incubated with 2 mM NAC for 12 h and then additionally treated with 8 μM LA-UC or 15 μM CCCP for 12 h. (**A**) MTT assay was performed to determine cell viability. (**B**) Mitochondrial ROS levels were measured by MitoSOX^TM^ staining. Scale bar, 20 μm. (**C**) Mitochondrial membrane potential was evaluated by JC-1 staining. (**D**–**F**) Cells were treated the same as in (**A**–**C**), and cell lysates were analyzed by western blotting for endogenous proteins. Representative images are shown. The expression levels of proteins were normalized to GAPDH or actin, and then the fold change was calculated by dividing each sample’s value by the control value. Results are presented as mean ± standard error of the mean (SEM) of triplicate independent experiments. * *p* < 0.05, ** *p* < 0.01, and *** *p* < 0.001 compared to control. C-Cas3: cleaved caspase-3; Cyt C: cytochrome C.

To examine whether LA-UC-induced mitochondrial ROS generation was associated with LA-UC-induced G2/M phase cell cycle arrest and caspase-dependent apoptosis, B16F10 cells were treated with LA-UC in the presence or absence of N-acetylcysteine (NAC). NAC treatment decreased the LA-UC-induced cell death of B16F10 cells (Figure 6A). In addition, NAC diminished the LA-UC-induced increase in mitochondrial ROS and impairment of mitochondrial membrane potential (Figure 6B,C), as well as the effects of LA-UC on key regulatory proteins related to G2/M phase arrest and apoptosis, cyclin B1, Cdc2, Bak, Bim, cytochrome C, caspase-3, and γ-H2AX (Figure 6D–F). These findings indicate that excessive generation of mitochondrial ROS by the treatment of LA-UC is associated with LA-UC-induced G2/M phase DNA damage, cell cycle arrest, and caspase 3-dependent apoptosis.

## 3. Discussion

In this study, we isolated lucknolide A (LA) from a marine *Streptomyces* sp. strain 151KO-065 and confirmed its structure through detailed analysis of NMR and MS data. Despite its unique structure, LA exhibited no significant anti-tumor activity in our study. To develop its biological activity, we introduced a 10-undecenoyl group at the primary alcohol of LA, generating a new derivative LA-UC, via semi-synthesis. LA-UC preferentially inhibited the proliferation of melanoma cells, such as A375 and B16F10, suggesting that the 10-undecenoyl group plays a critical role in its anti-tumor activity. Further study on the chemical modification of LA is needed to evaluate and optimize the potency and specificity of LA-UC.

Our finding revealed that LA-UC exerts minimal effects on normal melanocytes or other tumor cell types, indicating its selective action against melanoma cells (Figure 2). Treatment with LA-UC triggered excessive mitochondrial ROS generation and mitochondrial membrane impairment in melanoma cells, which were associated with genomic DNA damage-mediated melanoma cell death. In A375 human melanoma cells, we also observed LA-UC-induced mitochondrial ROS production, mitochondrial membrane potential loss, and DNA damage. Treatment with NAC effectively mitigated these LA-UC-induced effects, consistent with observations in B16F10 cells (Supplementary Figure S8 A–D). These results strongly support the conclusion that LA-UC induces cell death in melanoma cells through mitochondrial ROS-dependent mechanisms.

Melanoma cells are known to exhibit high basal levels of ROS compared to keratinocytes and fibroblasts because of dysregulated mitochondrial activity, oncogenic mutations, and external factors such as UV radiation [28,29]. In melanoma, ROS play a dual role: they contribute to tumor development and progression while also influencing therapeutic responses [30]. Elevated ROS generation in melanoma cells causes DNA damage, leading to mutations in oncogenes such as *B-Raf* and *N-RAS*. Additionally, elevated ROS levels activate several key signaling pathways, including mitogen-activated protein kinase (MAPK), phosphoinositide 3-kinase (PI3K), and protein kinase B (AKT). These pathways promote aggressive cell proliferation, survival, resistance to apoptosis, and an increased risk of carcinogenesis [31,32,33]. To counteract elevated ROS levels, melanoma cells also upregulate their antioxidant systems, such as glutathione, GSH, and catalase, which maintain ROS at sub-lethal levels and protect the cells from elevated ROS despite oxidative stress, contributing to the evasion of apoptosis and treatment resistance [34]. Therefore, while moderately high ROS levels promote melanoma survival and progression, excessive ROS levels lead to oxidative stress and melanoma cell death. Given this dual role of ROS in melanoma, regulating ROS levels may be a key therapeutic strategy for melanoma treatment. Targeting mitochondrial ROS may be particularly promising for melanoma cells with high mitochondrial metabolic activity [35]. This approach could serve as an effective anticancer strategy aimed at mitochondrial dysfunction, thereby preferentially inducing apoptosis in melanoma cells while minimizing harm to other types of cells.

Our study showed that LA-UC significantly increased mitochondrial ROS production, leading to mitochondrial membrane damage and dysfunction in B16F10 melanoma cells. This results in the release of pro-apoptotic factors such as cytochrome c, which initiates the intrinsic apoptotic pathway. Therefore, these findings suggest that LA-UC is a promising therapeutic candidate for tumors with mitochondrial oxidative vulnerabilities, such as melanomas, and may offer precise and effective treatment methods with fewer side effects than conventional therapies. Lastly, further studies are necessary to investigate the variability in sensitivity to the effects of LA-UC among different melanoma cell lines. Indeed, we observed that the effect of LA-UC on mitochondrial ROS-dependent cell death was significantly greater in B16F10 cells than in A375 cells. B16F10 cells exhibited higher sensitivity to LA-UC-induced mitochondrial ROS production, mitochondrial membrane disruption, and cell death compared to A375 cells. Furthermore, in A375 cells, treatment with NAC or Z-VAD only partially reduced the ROS-dependent effects induced by LA-UC, and, while the reduction was statistically significant, its extent remained modest. These differences may be due to variations in intracellular ROS levels or ROS regulator mechanisms between the cell lines. While mitochondrial ROS play a critical role in LA-UC-induced melanoma cell death, additional mechanisms may also contribute, particularly in A375 cells. Pathways such as mitochondrial ROS-independent or caspase-independent mechanisms could be involved. Further studies are required to fully elucidate these pathways and their relevance to LA-UC’s action in various melanoma cell lines. Such research could help to fully elucidate additional potential mechanisms of action for LA-UC, explain the differences in responses among cell types, and enable a more comprehensive evaluation of its therapeutic potential.

## 4. Materials and Methods

### 4.1. Chemicals and Reagents

The MTT was purchased from Sigma Chemical Co. (St. Louis, MO, USA). Z-VAD-FMK was purchased from Selleck Chemicals (Houston, TX, USA). Primary antibodies against Bak (#12105), Bim (#2933), caspase-3 (#14220), cleaved caspase-3 (#9664), caspase-9 (#9508), cytochrome C (#11940), and phospho-histone H2AX (#9718) were purchased from Cell Signaling Technology (Danvers, MA, USA). Cyclin B1 (sc-7393), Cdc2, p34 (sc-54), and GAPDH (sc-25778) were purchased from Santa Cruz Biotechnology (Santa Cruz, CA, USA), while actin (MAB1501) was purchased from Millipore (Billerica, MA, USA). All other chemicals and reagents were purchased from Sigma-Aldrich (Merck Millipore, unless stated otherwise).

### 4.2. Extraction and Isolation of LA

A marine actinomycete strain isolated from a mangrove sample collected in Kosrae Island was identified as *Streptomyces* sp. 151KO-065 by 16S rRNA gene sequence analysis. The strain 151KO-065 was grown on a Bennett’s (BN) agar plate for 7 days at 28 °C and then incubated in BN medium in a 50 mL flask. After 4 days of cultivation at 28 °C, 10 mL of the seed culture was used to inoculate 1 L of BN medium in a 2 L flask for 4 days. For a large-scale culture, 1 L of the culture in a 2 L flask was used to inoculate 60 L of BN medium in a 100 L fermenter. The large-scale culture was incubated at 28 °C for 7 days. The culture broth (60 L) was separated into the cells and the supernatant by continuous centrifugation. The supernatant was extracted with EtOAc (60 L × 2) at room temperature and then concentrated in vacuo to yield a crude extract (10 g). The crude extract was fractionated by flash column chromatography on ODS using a stepwise elution with combinations of MeOH/H_2_O (1:4, 2:3, 3:2, 4:1, and 100% MeOH). The fraction eluted with MeOH/H_2_O (1:4) was repeatedly chromatographed with 10% MeOH, 15% MeOH, and 20% MeOH in H_2_O. The 15% MeOH subfraction was further purified via C18 reversed-phase HPLC (YMC-Pack-ODS-A, 250 × 10 mm i.d., 5 µm particle size, flow rate: 4.0 mL/min, RI detector) under an isocratic condition with 10% MeOH in H_2_O, resulting in the isolation of lucknolide A (125 mg).

### 4.3. Semi-Synthesis of LA-UC

To synthesize LA-UC, LA (20 mg, 0.088 mM) was dissolved in 2 mL of pyridine as a solvent. 10-Undecenoyl chloride (21 µL, 0.106 mM, 1.2 equivalents) was added dropwise under an inert atmosphere, and the reaction mixture was stirred at room temperature overnight (12–16 h). After the reaction was completed, the mixture was diluted with water and extracted with ethyl acetate. The combined organic layers were washed with 1 M HCl, saturated sodium bicarbonate solution, and brine, then dried over anhydrous sodium sulfate. The solvent was removed under reduced pressure, and the crude product was purified via open column chromatography (silica gel) using a gradient elution of hexane/ethyl acetate, yielding LA-UC as a white solid.

### 4.4. Cell Culture and MTT Assay

The B16F10 mouse melanoma cells and A375 human melanoma cells were obtained from the Korean Cell Line Bank (KCLB, Seoul, Republic of Korea) and cultured in Dulbecco’s modified Eagle medium (Welgene, Gyeongsangbuk-do, Republic of Korea) containing 10% fetal bovine serum (FBS, Welgene, Republic of Korea), 100 U/mL of penicillin, and 100 μg/mL of streptomycin in a 5% CO_2_ incubator at 37 °C. Primary epidermal melanocytes (normal, human, adult; HEMa) and their corresponding growth medium were obtained from ATCC (American Type Culture Collection, Manassas, VA, USA). The anti-growth effect of LA-UC was measured using the MTT assay. B16F10 cells were seeded at a density of 4 × 10^3^ cells/well in 96-well plates. After 12 h of seeding, the cells were incubated with vehicle dimethyl sulfoxide (DMSO) or various concentrations of LA-UC for 24 h. Subsequently, the cells were treated with the MTT solution (1 mg/mL final concentration) and incubated for 4 h at 37 °C. The resulting formazan crystals were dissolved in 100 μL DMSO. The absorbance was determined at 595 nm using a microplate reader (FilterMax F5, Molecular Devices) (Molecular Devices, LLC Sunnyvale, CA, USA).

### 4.5. Apoptosis Assay (Fluorescence-Activated Cell Sorting [FACS]: Flow Cytometry)

The cells were seeded at a density of 5 × 10^4^ cells/well in a 12-well plate. After incubation for 12 h, the cells were treated with vehicle DMSO or LA-UC for 24 h. The cells were washed twice with phosphate-buffered saline (PBS), adjusted to 100 μL of solution, and stained with annexin fluorescein isothiocyanate-conjugated annexin V (V-FITC) and PI using the FITC Annexin V Apoptosis Detection Kit (BD Biosciences, San Jose, CA, USA). The cells were incubated for 10 min at room temperature. The cells were analyzed using a FACSVerse^TM^ flow cytometer (BD Bioscience, Franklin Lakes, NJ, USA) (Ex/Em = 488 nm/530 nm), and the data were calculated using flowJo^TM^ v10.8.0 software (BD Bioscience, Franklin Lakes, NJ, USA).

### 4.6. Immunofluorescence

Cells were seeded on coverslips and treated with DMSO. LA-UC was washed once with ice-cold PBS and fixed with 4% paraformaldehyde for 5 min at room temperature. After fixation, the cells were washed thrice with PBS and permeabilized with 0.1% *v*/*v* Triton X-100 for 5 min, followed by three additional washes with PBS. A PBS-blocking solution containing 1% bovine serum albumin was added to the coverslips. After incubation for 30 min, appropriately diluted primary and secondary antibodies were added, followed by incubation for 1 h at room temperature. The coverslips were washed thrice with PBS and mounted on glass slides using a Pro-Long Gold anti-fade mounting medium (Invitrogen, Waltham, MA, USA). The coverslips were imaged using a Zeiss LSM780 confocal microscope (Zeiss LSM 780, Oberkochen, Germany) with a 40× oil-free immersion objective.

### 4.7. Mitochondrial Membrane Potential Assay

The mitochondrial membrane potential was determined using a JC-1 kit (BD Bioscience, Franklin Lakes, NJ, USA). The cells (4 × 10^3^ cells/well) were seeded in 18-well plates and treated with LA-UC for 12 h. The cells were incubated with JC-1 at 37 °C for 20 min and washed twice with 1 × assay buffer. Changes in mitochondrial membrane potential were detected using a Zeiss LSM780 confocal microscope (Zeiss LSM 780, Oberkochen, Germany) with a 40× oil-free immersion objective.

### 4.8. ROS Detection

To detect mitochondrial ROS levels, cells (4 × 10^3^ cells/well) were seeded in 18-well plates and treated with LA-UC for 12 h. The cells were incubated with MitoSOX^TM^ reagent at 37 °C for 20 min and washed twice with 1 × assay buffer. The ROS levels were detected using a Zeiss LSM780 confocal microscope (Zeiss LSM 780; Oberkochen, Germany) with a 40× oil-free immersion objective.

### 4.9. Western Blot Analysis

Western blotting was performed according to standard procedures. Briefly, B16F10 cells (2 × 10^5^ cells/well) were seeded in six-well plates in a serum-free medium. After incubation for 24 h, the cells were treated with vehicle DMSO, LA-UC, or other compounds for 24 h. The cells were lysed in 2× sample buffer at 4 °C. Total protein was extracted using 8–10% sodium dodecyl sulfate sulfate-polyacrylamide gels and transferred to polyvinylidene difluoride membranes (Amersham Pharmacia Biotech, England, UK). All proteins were quantified as housekeeping proteins such as GAPDH and actin. The reaction was blocked with 5% skim milk and 1% bovine serum albumin in PBS for 1 h at RT and probed with primary and secondary antibodies. The proteins were detected using a chemiluminescent ECL assay kit (Amersham Pharmacia, Buckinghamshire, UK). Immunoblotted bands were visualized using a LAS-3000 system and quantified using MultiGauge V 3.0 software (Fujifilm Life Science, Tokyo, Japan).

### 4.10. Statistical Analysis

All data were analyzed using the Instat statistics program (GraphPad version 5.01 Software, Inc., San Diego, CA, USA). Statistical comparisons were performed using the *t*-test. All results are presented as mean ± standard error of at least three independent experiments. * *p* < 0.05, ** *p* < 0.01, *** *p* < 0.001 compared to the control.

## 5. Conclusions

In this study, we isolated lucknolide A (LA) from a marine-derived *Streptomyces* sp. and synthesized its derivative, LA-UC, through semi-synthesis to enhance its biological activity. While LA showed no significant anti-tumor effects, LA-UC exhibited potent anti-tumor activity by preferentially inhibiting the proliferation of melanoma cells, including B16F10, while exerting minimal effects on normal melanocytes or other tumor cell types. This indicates the selective action of LA-UC against melanoma cells. Our results show that LA-UC functions as a mitochondrial ROS-inducing agent and an effective G2/M arrest- and apoptosis-inducing agent in melanoma B16F10 cells. Specifically, LA-UC induced excessive mitochondrial ROS production and impaired mitochondrial membrane integrity, leading to genomic DNA damage, G2/M phase cell cycle arrest, and caspase-dependent apoptosis in B16F10 cells. Given its ability to selectively target melanoma cells through mitochondrial oxidative stress, LA-UC is a promising therapeutic candidate for treating tumors with mitochondrial oxidative vulnerability, such as melanoma.

## Data Availability

The data that support the findings of this study are available from the corresponding author (H.J.S. and S.J.P.) upon reasonable request.

## Supplementary Materials

The following supporting information can be downloaded at: https://www.mdpi.com/article/10.3390/md22120533/s1, Figure S1: ESIMS data of LA; Figure S2: ^1^H and ^13^C NMR spectra of LA; Figure S3: HSQC spectrum of LA; Figure S4: ^1^H-^1^H COSY spectrum of LA; Figure S5: HMBC spectrum of LA; Figure S6: ESIMS data of LA-UC; Figure S7: ^1^H and ^13^C NMR spectra of LA-UC; Figure S8: Data of LA-UC in A375 cells.
