# Supplementary material for: A Lucknolide Derivative Induces Mitochondrial ROS-Mediated G2/M Arrest and Apoptotic Cell Death in B16F10 Mouse Melanoma Cells
Source: Mar Drugs. 2024 Nov 28;22(12):533. doi: 10.3390/md22120533 (PMC11677583; doi:10.3390/md22120533)

Supplementary Figure S1. ESIMS data of LA.

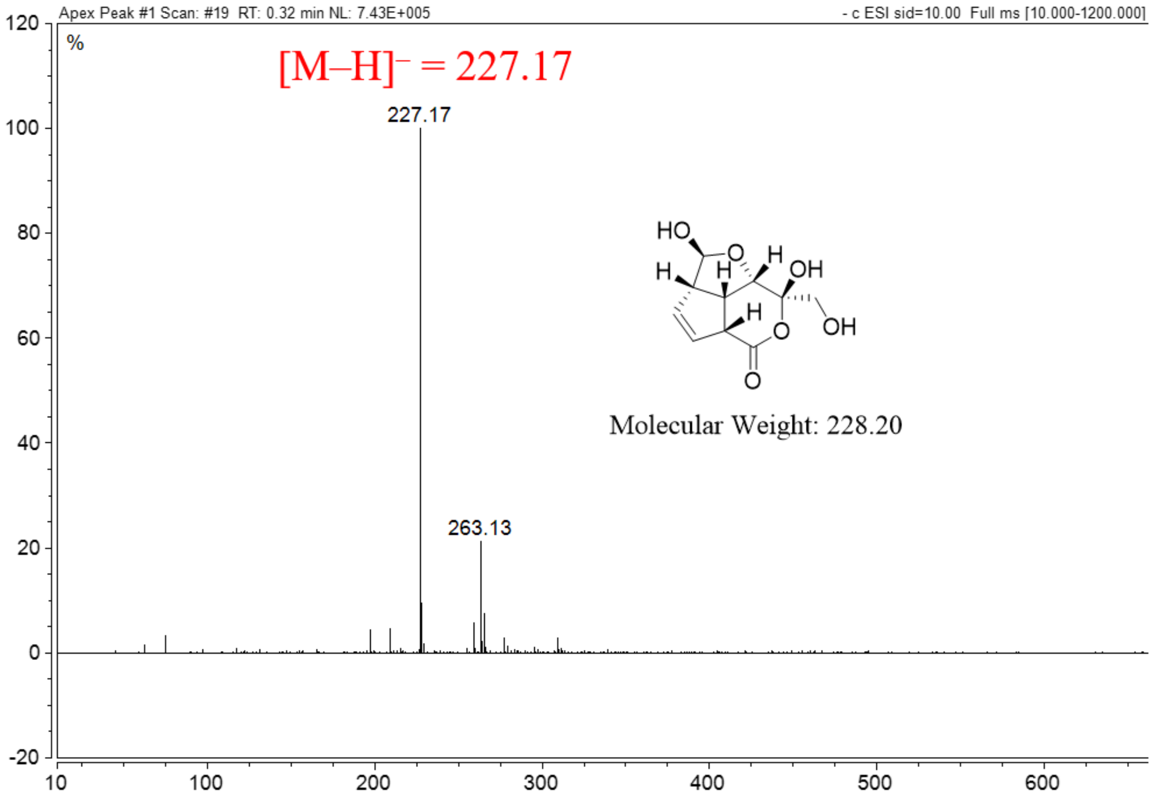

Supplementary Figure S2. ^{1}H and ^{13}C NMR spectra of LA.

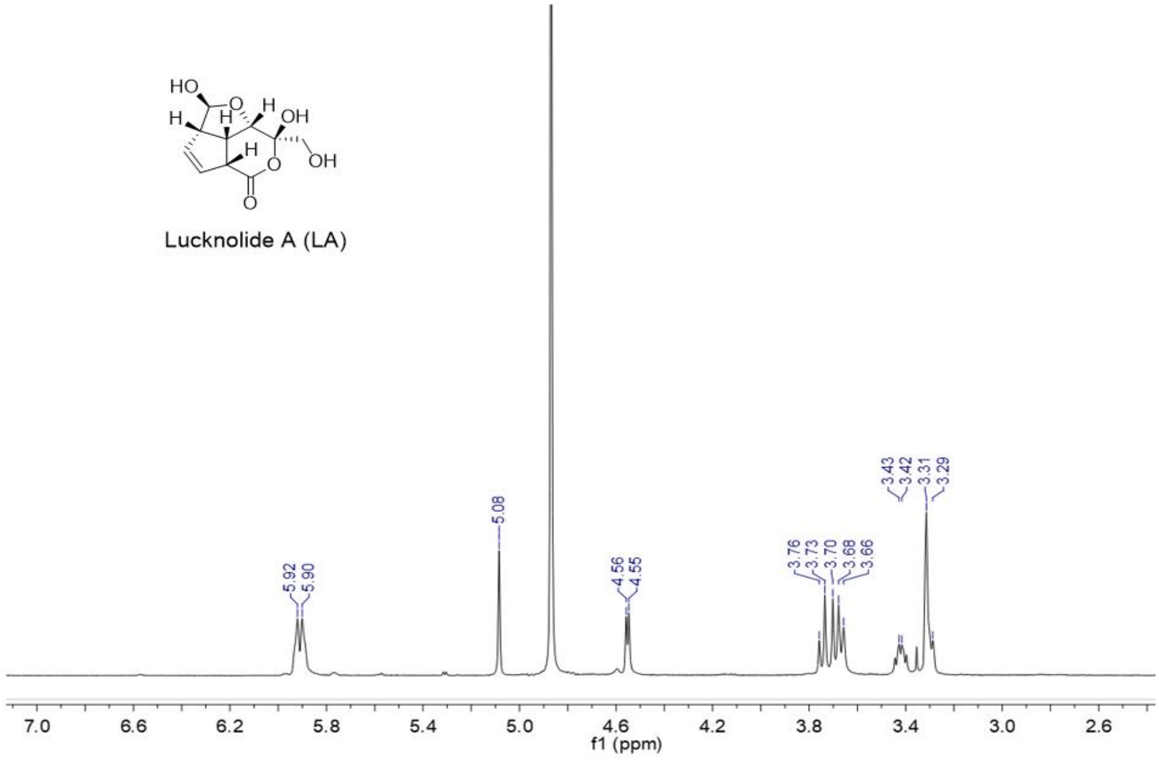

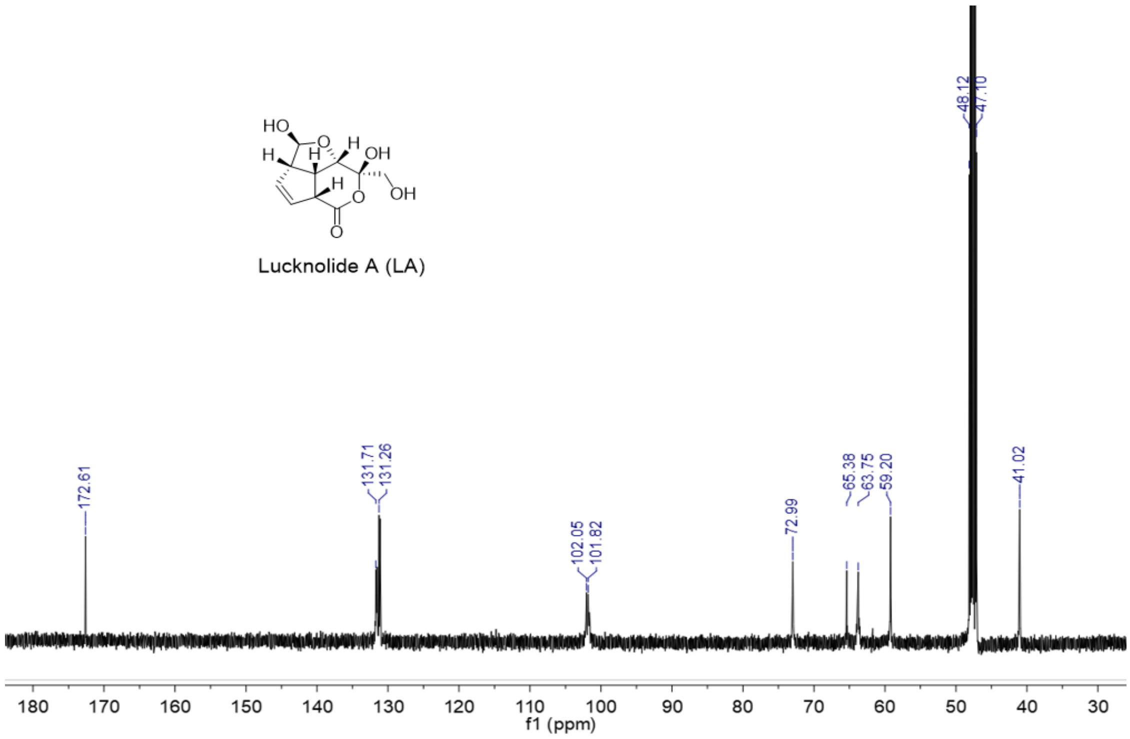

Supplementary Figure S3. HSQC spectrum of LA.

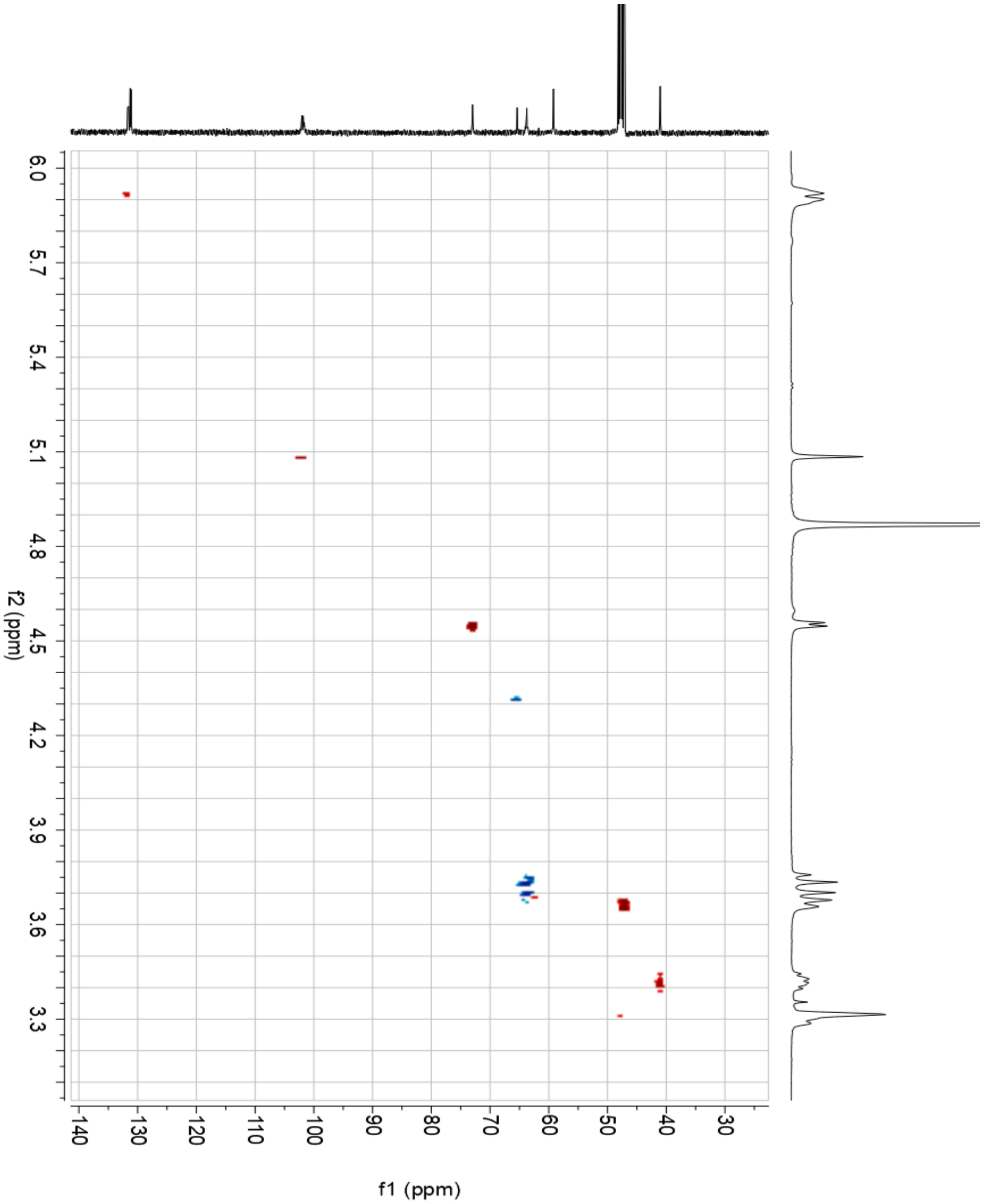

Supplementary Figure S4. ^1H-^1H COSY spectrum of LA.

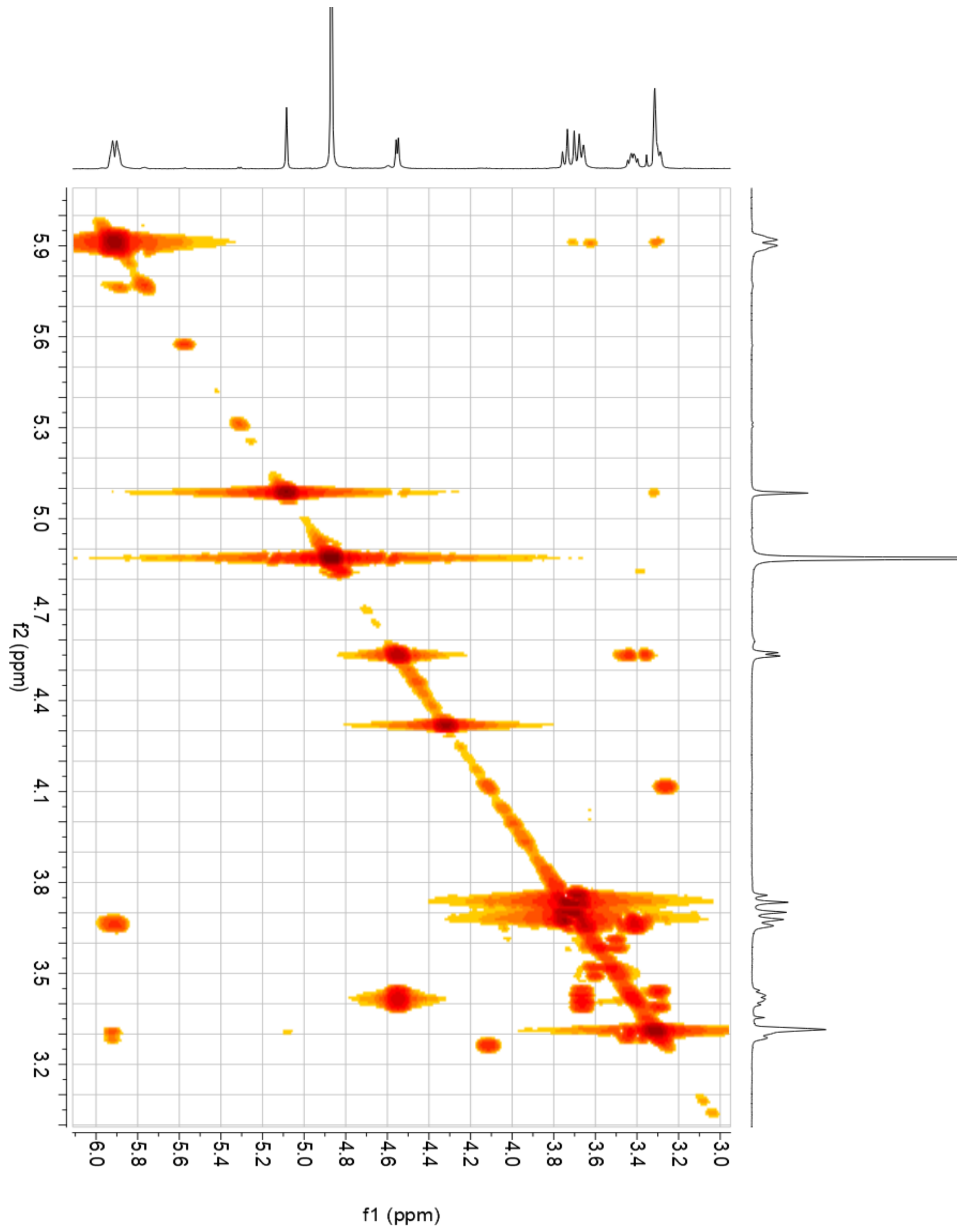

Supplementary Figure S5. HMBC spectrum of LA.

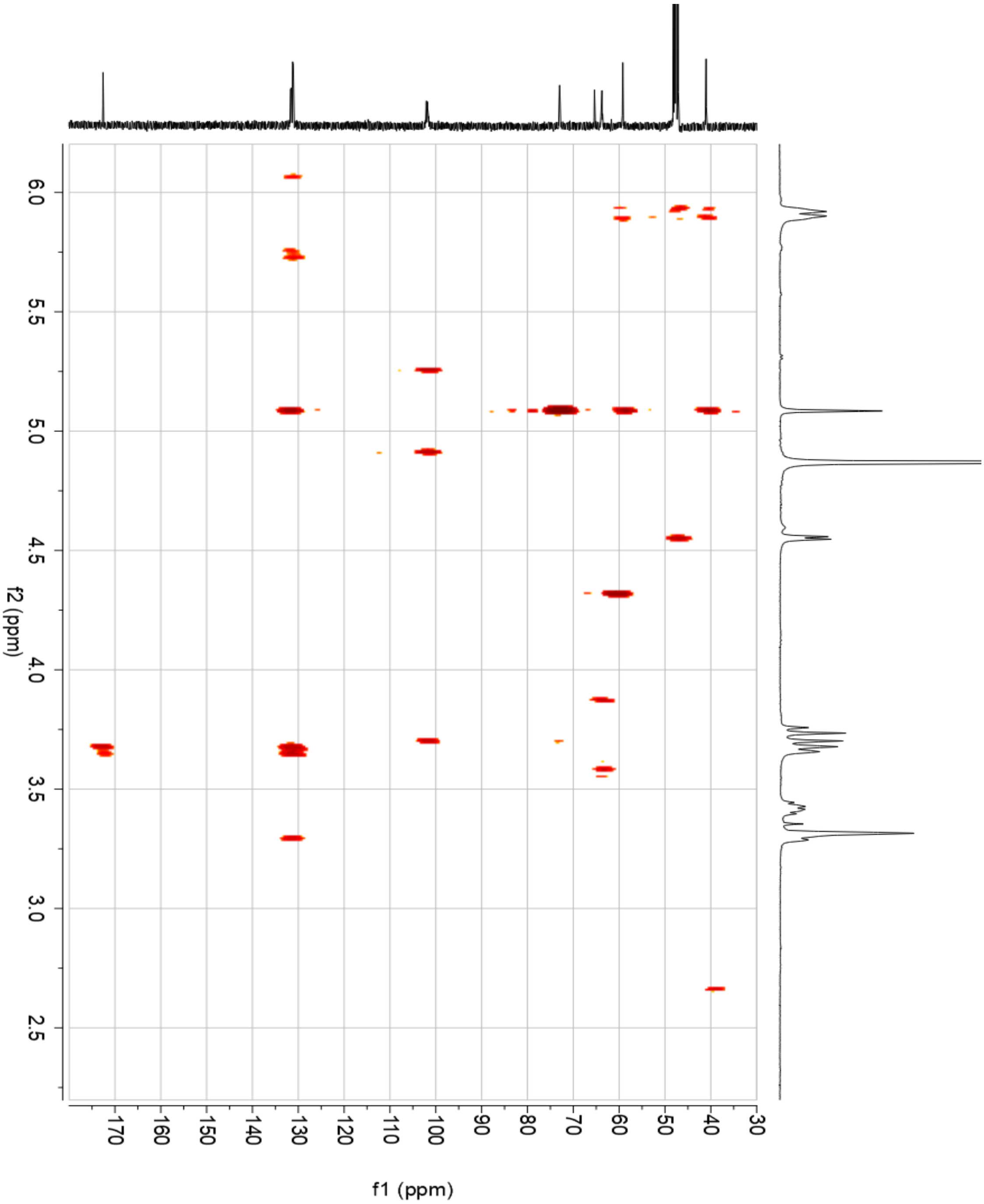

Supplementary Figure S6. ESIMS data of LA-UC.

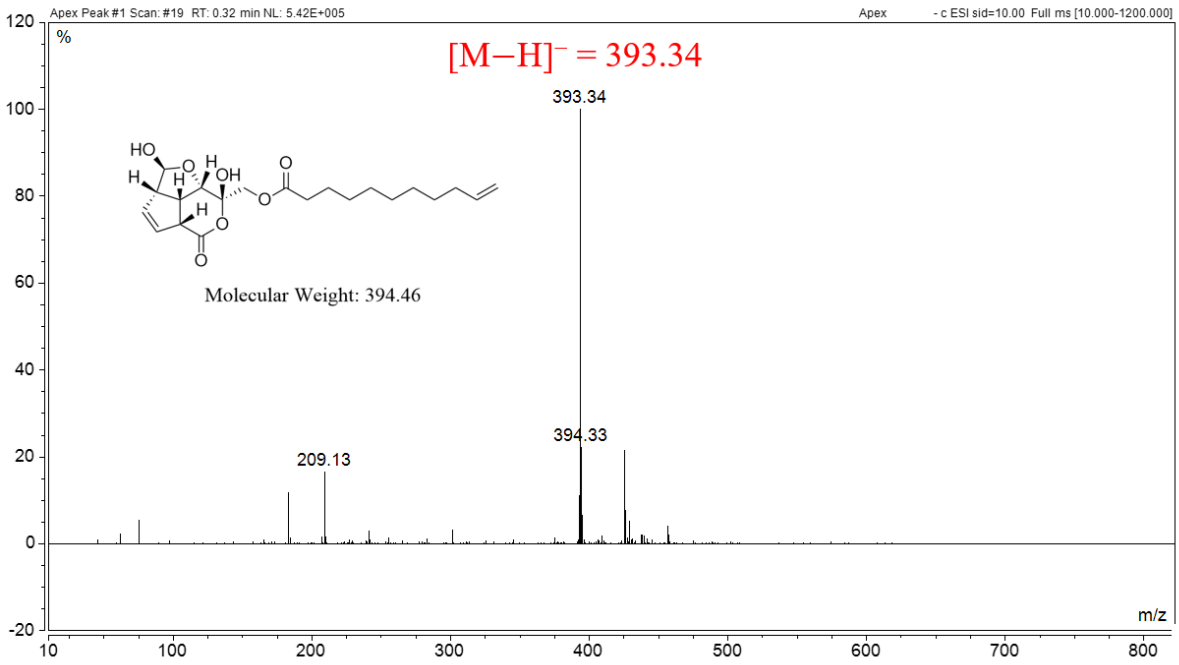

Supplementary Figure S7. ^{1}H and ^{13}C NMR spectra of LA-UC.

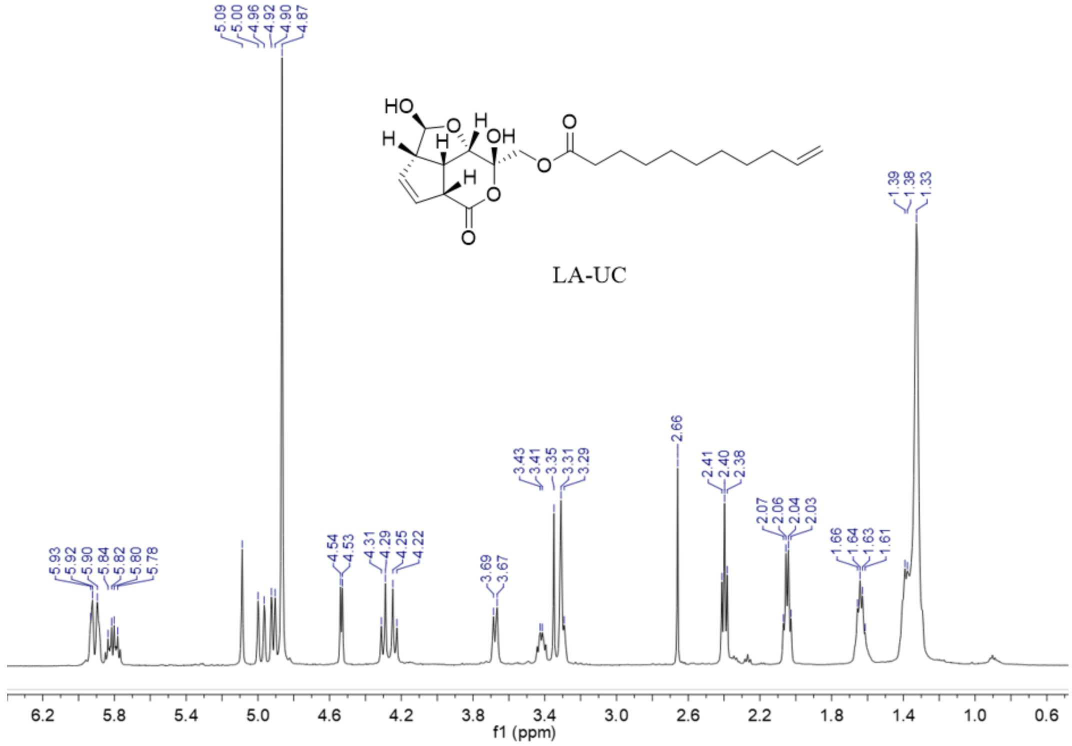

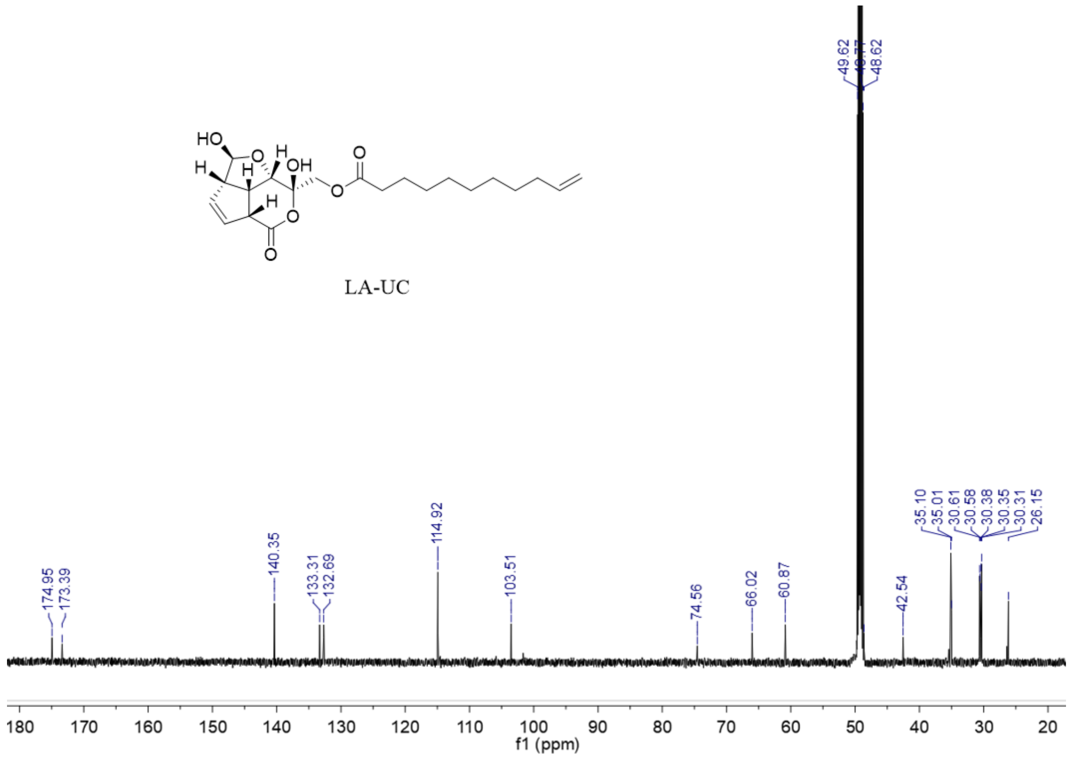

Supplementary Figure S8. LA-UC in A375 cells

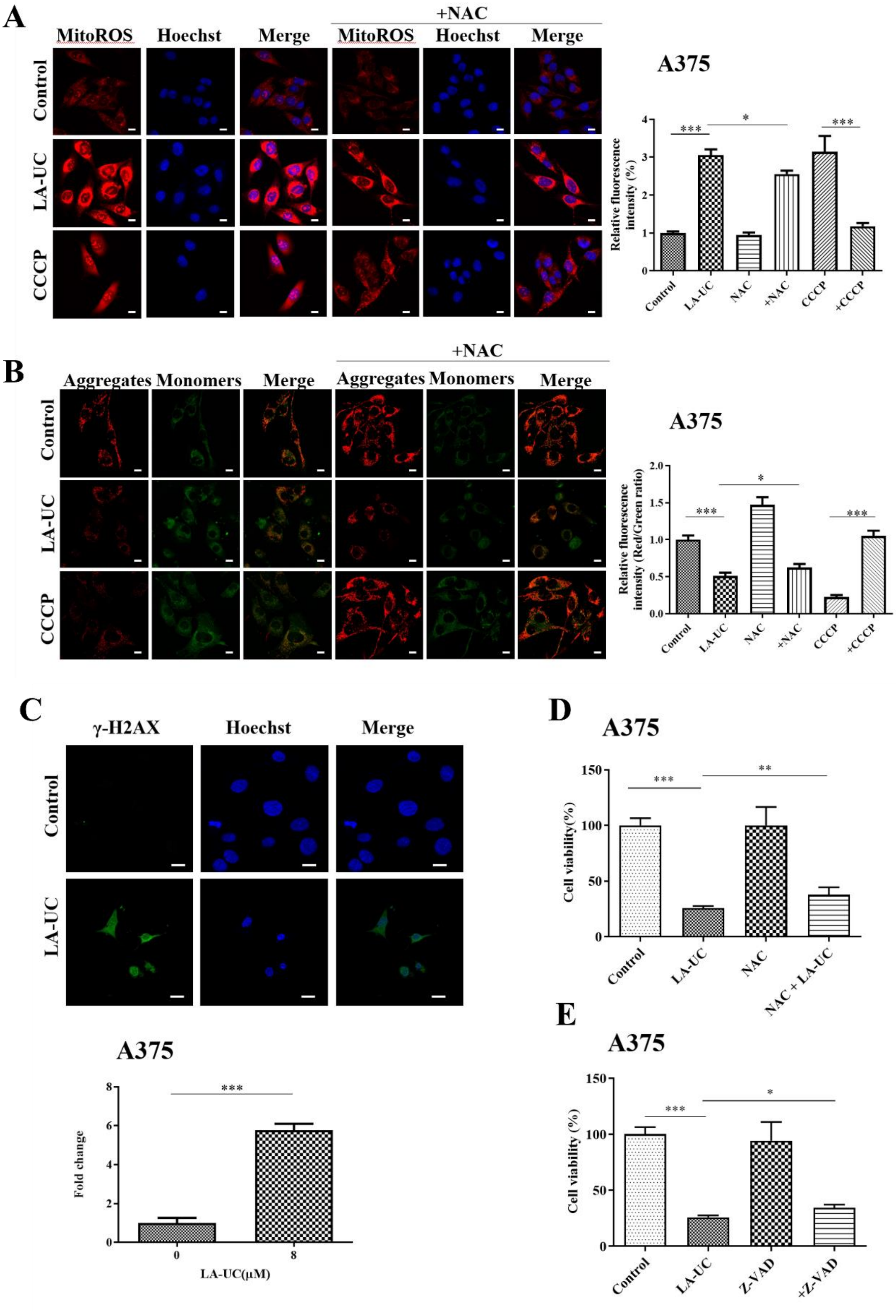

Supplement: Supplementary file 1 [file marinedrugs-22-00533-s001.zip › marinedrugs-3309581-supplementary.pdf]
